# Baricitinib for the Management of SARS-CoV-2-Infected Patients: A Systematic Review and Meta-Analysis of Randomised Controlled Trials

**DOI:** 10.1155/2022/8332819

**Published:** 2022-08-02

**Authors:** Sivananthan Manoharan, Lee Ying Ying

**Affiliations:** ^1^Molecular Pathology Unit, Cancer Research Centre, Institute for Medical Research, National Institutes of Health, Ministry of Health Malaysia, Setia Alam 40170, Shah Alam, Selangor, Malaysia; ^2^Asia Metropolitan University, Bandar Baru Seri Alam 81750, Johor Bahru, Johor, Malaysia

## Abstract

Baricitinib is known to reduce mortality and disease progression in COVID-19 patients; however, the data are inconsistent. Therefore, it needs to be explored to further understand the clinical benefits of this drug in the management of COVID-19 patients. Does baricitinib statistically significantly reduce mortality and disease progression in COVID-19 patients? To answer these questions, three databases known as ScienceDirect, PubMed/MEDLINE, and Scopus and other sources, such as preprint (medRxiv) and reference lists, were thoroughly searched. Four randomised controlled trials (RCTs) were included. Based on the meta-analysis, baricitinib statistically significantly reduced mortality with the risk ratio (RR) of RR = 0.74 [95% CI: 0.58 to 0.94; *p* = 0.01] and moderately high heterogeneity, where *I*^2^ = 62% and *p* = 0.05. On the other hand, RR = 0.84 [95% CI: 0.75 to 0.95; *p* = 0.005] with insignificant heterogeneity of *I*^2^ = 20% and *p* = 0.28 was found for disease progression. Cochrane risk of bias (RoB) analysis revealed that three out of four articles were ranked as high-quality articles with low RoB. Based on the evidence grading, the overall certainty of evidences was moderate. In conclusion, baricitinib statistically significantly reduced mortality and disease progression in COVID-19 patients when the patients were treated with baricitinib at a dosage of 2 mg or 4 mg for a maximum duration of 14 days.

## 1. Introduction

As of 27^th^ May 2022, more than 530 million people were infected with the novel SARS-CoV-2, which caused COVID-19. More than 6.3 million deaths were recorded, making COVID-19 as one of the deadly diseases in human history [1]. According to Rochelle Walensky, the Director of Centers for Disease Control and Prevention (CDC), the delta strain of SARS-CoV-2/COVID-19 is one of the most infectious respiratory illnesses witnessed by specialists/experts. This strain is very contagious and aggressive compared to other SARS-CoV-2 viral strains [2]. Scientists were looking at several strategies to discover treatments for COVID-19. One of the strategies was to repurpose an anti-rheumatoid arthritis drug (baricitinib) for the management of COVID-19 patients. It is worth citing that on 14 January 2022, the World Health Organization (WHO) strongly endorsed the use of baricitinib with corticosteroids in severe or critical SARS-CoV-2-infected patients. In May 2022, the Food and Drug Administration (FDA) gave full approval to use baricitinib in adult COVID-19 patients and emergency use authorisation (EUA) for children between 2 and 18 years of age [3, 4]. Two research outputs from phase 3 randomised controlled trials (RCTs) led to the current approval of baricitinib by FDA [4]. RCT is regarded as a gold standard study design to explore the efficacy of treatments. When looking at the pyramid of study designs to collect evidence for any treatment effect, RCTs and meta-analyses (MAs) of RCTs are ranked at the highest of the pyramid and produce fairly good evidences. On the other hand, case reports are ranked at the bottom of the pyramid. MA can also be conducted using non-RCT (nRCT)-related evidences. However, MA based on nRCTs provides less strong evidences than MA of RCTs. Observational studies are complementary to RCTs [5]. According to the Cochrane Handbook for Systematic Reviews of Interventions version 6.3 (updated in February 2022), nRCTs are only included to MA when there are insufficient RCTs to answer the research question [6]. In the current systematic review (SR) and MA, the authors conducted SR and MA based on available RCTs for baricitinib. Despite the recent approvals by WHO and FDA, the outcomes from several RCTs related to baricitinib were conflicting. To the best of the authors' knowledge, this is the first SR and MA for baricitinib, which involves the inclusion of only RCT evidences. Furthermore, the authors contacted Marconi et al. [7] to acquire more data, especially related to invasive mechanical ventilation (IMV), which could not be found in other articles. In the current study, the nRCTs' results were not pooled together with RCTs to avoid merging different level of risk of bias (RoB).

## 2. Methodology

In the present SR and MA, the Preferred Reporting Items for Systematic Reviews and Meta-Analyses (PRISMA) guidelines were referred to build this review. These guidelines were adhered to accordingly. No advanced protocol related to the present SR and MA was developed or registered.

### 2.1. Research Questions

1. What is the statistical ability of baricitinib to reduce mortality/death in COVID-19 patients?
2. What is the statistical ability of baricitinib to reduce disease progression in COVID-19 patients?

### 2.2. Search Strategies, Article Eligibility Criteria, and Data Charting Process

Three main databases, known as PubMed/MEDLINE, ScienceDirect, and Scopus, and other sources, such as preprint (medRxiv) and reference list, were searched thoroughly using keywords, namely, “Randomised controlled trials baricitinib COVID-19; Randomised controlled trials baricitinib SARS-CoV-2 virus; and Randomised controlled trials baricitinib pneumonia.” The searched period was between 2020 and March 2022. The inclusion criteria were as follows:

- Patients infected with SARS-CoV-2
- Use of baricitinib as the drug for intervention purposes
- Presence of proper control/s
- Strictly only for RCT studies
- Clinical efficacy stated in the study outcomes
- Only articles in the English language

The data charting activities included screening of titles, abstracts, and texts, which were undertaken completely by two authors, independently.

### 2.3. Risk of Bias Analysis (RoB) and Grading the Evidence with GRADEpro and Conduct of MA

The Cochrane RoB tool was deployed to measure the quality of RCTs [8]. Furthermore, the authors graded the quality of the articles using GRADEpro Guideline Development Tool software [9]. The MA was carried out using Review Manager 5.4.1 [8]. Dichotomous data type was used. The RCT data were pooled, and relative risk, confidence interval (CI), and Mantel–Haenszel statistical approaches were used. The degree of heterogeneity was assessed based on *p* value, the *I*^2^ test. Heterogeneity was defined as significant when *p* < 0.1 or *I*^2^>50% [10]. A fixed-effect model was used when the data were homogeneous, while a random-effect model was used if the data were heterogeneous. The publication bias (PB) was not checked since only four articles were available. At least ten articles are required to produce a good statistical related funnel plot for detection of PB.

## 3. Results and Discussion

**Figure 1:**
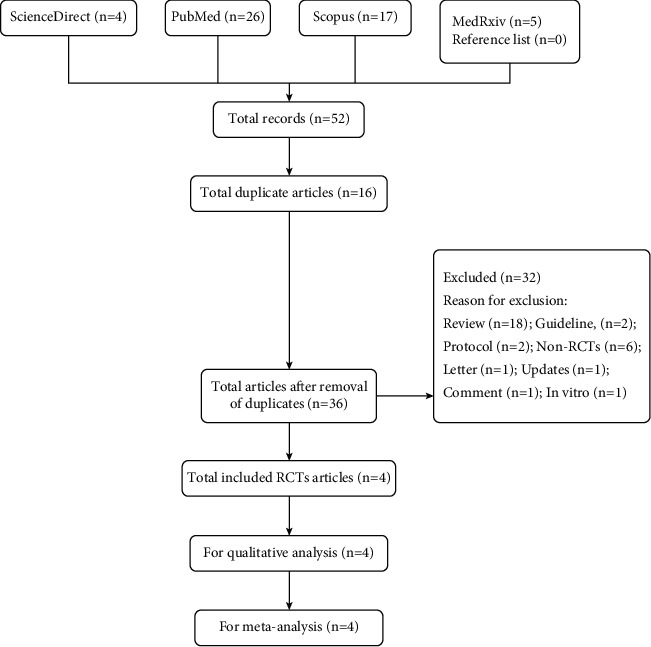
The flowchart detailing the systematic literature search. The chart was prepared using Review Manager 5.4.1 [8].

**Figure 2:**
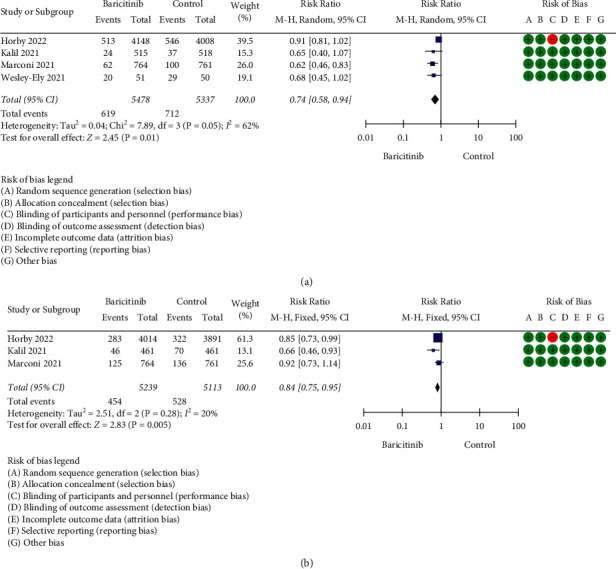
(a) The effect of baricitinib on mortality. (b) The effect of baricitinib on disease progression in COVID-19 patients. Marconi et al. [7] were contacted for additional information. The additional information was supplied by Eli Lilly, the sponsor company. The letter is supplied as supplementary file S2.

Based on strict literature search according to the keywords, a total of 52 studies were found and four full RCTs were included for qualitative (SR) and quantitative (MA) analyses (Figure 1). All searched articles could be found in the supplementary file S1. Only four RCTs were available within the search period (up to March 2022); however, the inclusion of more than 10,000 sample size (RCTs' participants) represented the total population of more than 300 million [11]. In fact, according to Eli Lilly and Company (Lilly), so far about one million patients received baricitinib for the management of COVID-19 in 15 countries [4]. In this scenario, although four RCTs were included, the cumulative sample size, which was more than 10,000 participants, represented a large number of human populations.  Table 1 shows the characteristics of the included studies. Except for a study by Horby et al. [12], all other three RCTs were found to be low in RoB. The blinding strategy by Horby et al. [12] was weak due to the open-label study design. Due to the weak blinding strategy, based on the evidence grading through certainty assessments, the overall certainty of the pooled RCTs was moderate (Table 2). Moreover, based on MA for mortality, with the inclusion of 10,815 sample size, baricitinib statistically significantly reduced the mortality in COVID-19 patients with the risk ratio (RR) of RR = 0.74 [95% CI: 0.58 to 0.94; *p* = 0.01], but moderate high heterogeneity of *I*^2^ = 62% and *p* = 0.05 was found (Figure 2(a)). The heterogeneity was suspected to be due to a large sample size by Horby et al. [12]. A large sample size is required to reflect the actual effect of baricitinib in COVID-19 patients. The authors speculated that although it was feasible, it was difficult to conduct blinding strategy when the sample size is extremely large. However, subgroup analysis was not conducted with the inclusion of only low RoB articles due to the fact that the bigger the sample size, the better the detection of actual effect of the drug in COVID-19 patients. Besides, findings by Horby et al. [12] were related to a large sample size (2/3 of total size) and could not be simply excluded. The individual value of RR (0.62–0.91) for all 4 studies supporting baricitinib's efficacy in reducing mortality in COVID-19 patients.

On the other hand, baricitinib statistically significantly reduced disease progression in COVID-19 patients with RR = 0.84 [95% CI: 0.75 to 0.95; *p*=0.005], but insignificant heterogeneity of *I*^2^ = 20% and *p*=0.28 was found (Figure 2(b)). The data related to IMV were used for this analysis because the progression of COVID-19 patients from early/mid-stage to later stage, especially requiring IMV, is regarded as an extremely worsening health condition in COVID-19 patients. King et al. [15] reported that high mortality rate was seen in COVID-19 patients requiring IMV, especially in older patients. According to Goletti and Cantini [16], while remdesivir is appropriate for early stage of COVID-19 management and corticosteroid is fit for the advanced stage of COVID-19 treatment, baricitinib could be utilised in between these two drugs to stop moderately severe COVID-19 patients from encountering the later stage. Due to the importance of IMV data, the authors contacted Marconi et al. [7] to acquire more detailed information mainly related to IMV since this information was not directly available in the article. There was no response from Kalil et al. [13] to gather similar data; therefore, the authors continued with the available IMV/ECMO data. This could serve as a confounding factor; however, it was considered that COVID-19 patients requiring extracorporeal membrane oxygenation (ECMO) were probably lesser than those requiring IMV. This is because ECMO is a costly medical device and probably only available for very limited number of patients. In the current analysis, based on 10,352 sample size, it was proven that baricitinib was able to reduce the disease progression related to IMV in COVID-19 patients. Based on both analyses, baricitinib reduced mortality and disease progression by 13.1% and 14%, respectively.

The advantages of the current MA compared to other MAs indicated the following: (i) large sample size was included to derive concrete outcomes, (ii) only RCT studies were included, (iii) the focus for block disease progression was IMV, (iv) communication was established with one corresponding author to gather more data related to IMV, which was supplied by Lilly, the sponsor company, and (v) GradePro tool was used for grading the evidences, whereby this strategy was lacking in other MAs related to baricitinib. The limitation of the current study was that the result should be interpreted carefully since the heterogeneity was moderately high for mortality. Furthermore, due to the open-label study, performance bias was identified in a study by Horby et al. [12]. This bias could compromise the overall findings, moderately. The authors were unable to exclude this study because the sample size was very large, and more than 99% of the participants were properly followed up. Furthermore, it was considered that this study was probably close to reality due to the broad inclusion criteria, whereby the patients were properly randomised and the outcome data were masked. Understanding this situation, Horby et al. [12] stated that despite the open-label study, the outcomes were clear-cut and were established without bias via linkage to routine health records. Besides, in the article, the authors mentioned that although the participants and study staff were unmasked (open-label/non-blind), the trial committee, investigators, and individuals involved in the study (other than participants and study staff) were masked (blinded) to the trial data outcome.

## 4. Conclusion

In conclusion, baricitinib statistically significantly reduced mortality and disease progression in COVID-19 patients when the patients were treated at the dosage of 2 mg or 4 mg for a maximum duration of 14 days.

## Figures and Tables

**Table 1:** Characteristics of the included articles and the outcome/s.

Study	Study design and phase	Period of study	Country	Population and age (*B* vs. *C*)	Intervention	Comparator	Outcomes	Cochrane RoB
Horby et al. [12]	RCTs-open label and platform trial (factorial design)	Feb 21–Dec 21	1 (UK)	>100 centers/hospitals (58.5 vs. 57.7)	(i) 4 mg baricitinib for 10 days (ii) Reduced dose in case of low eGFR (<60 mL/min) or patients taking probenecid or in children <9 years old	Usual care	Mortality: reduced IMV: not reduced	High (due to open-label study design)
Kalil et al. [13]	Full RCTs and phase III trial	May–July 20	8 countries	67 centers (55 vs. 55.8)	(i) Baricitinib 4 mg (maximum 14 days) with remdesivir (maximum 10 days) (ii) Baricitinib 2 mg (with other health related problems) (maximum 14 days) with remdesivir (maximum 10 days)	Remdesivir	Mortality: not reduced IMV/ECMO: reduced	Low
Marconi et al. [7]	Full RCTs and phase III trial	Jun 20–Jan 21	12 countries	>100 centers (57.8 vs. 57.5)	(i) Baricitinib 2 mg or 4 mg with matching SOC for maximum 14 days	Placebo with SOC	Mortality: reduced IMV: not reduced	Low
Ely et al. [14]	Full RCTs and phase III trial	Dec 20–Apr 21	4 countries	18 centers (58.4 vs. 58.8)	(i) Baricitinib 2 mg or 4 mg with SOC for maximum of 14 days	Placebo with SOC	Mortality: reduced IMV: NA	Low

IMV: invasive mechanical ventilation; SOC: standard of care; B: baricitinib; C: control; RoB: Risk of bias; UK: United Kingdom; eGFR: estimated glomerular filtration rate; ECMO: extracorporeal membrane oxygenation; NA: not available.

**Table 2:** Grading the evidence with GRADEpro guideline development tool.

Certainty assessment							No. of patients		Effect		Certainty	Importance
No of studies	Study design	Risk of bias	Inconsistency	Indirectness	Imprecision	Other considerations	Baricitinib	Control	Relative (95% CI)	Absolute (95% CI)		
*Mortality (4 RCTs)*												

4	Randomised trials	Serious^a^	Not serious	Not serious	Not serious	None	619/5478 (11.3%)	712/5337 (13.3%)	**RR 0.74** (0.58 to 0.94)	**35 fewer per 1,000** (from 56 fewer to 8 fewer)	**⊕⊕⊕ ◯** **Moderate**	Critical
*Disease progression (3 RCTs)*												

3	Randomised trials	Serious^a^	Not serious	Not serious	Not serious	None	454/5239 (8.7%)	528/5113 (10.3%)	**RR 0.84** (0.75 to 0.95)	**17 fewer per 1,000** (from 26 fewer to 5 fewer)	**⊕⊕⊕ ◯** **Moderate**	Critical

CI: confidence interval; RR: risk ratio. ^a^Open-label study [12].

## Data Availability

Most of the data for analysis are derived from published articles.

## Supplementary Materials

Supplementary Materials The supplementary files are given separately as supplementary files S1 and S2.
