# Supplementary material for: Baricitinib for the Management of SARS-CoV-2-Infected Patients: A Systematic Review and Meta-Analysis of Randomised Controlled Trials
Source: Can J Infect Dis Med Microbiol. 2022 Aug 2;2022:8332819. doi: 10.1155/2022/8332819 (PMC9346539; doi:10.1155/2022/8332819)
Supplement: Supplementary Materials — The supplementary files are given separately as supplementary files S1 and S2. [file 8332819.f1.zip › S1 file.pdf]

PubMed/Medli (Total n=26) (n=11 duplicates) (Total=15)	Reason for exclusion
A living WHO guideline on drugs for covid-19.	Guideline
An EUA for baricitinib (Olumiant) for COVID-19.	Letter
An update on drugs with therapeutic potential for SARS-CoV-2 (COVID-19) treatment.	Updates
Antirheumatic Disease Therapies for the Treatment of COVID-19: A Systematic Review and Meta-Analysis.	Review
Baricitinib and dexamethasone for hospitalized patients with COVID-19.	Non-RCTs
Clinical Management of Adult Patients with COVID-19 Outside Intensive Care Units: Guidelines from the Italian Society of Anti-Infective Therapy (SITA) and the Italian Society of Pulmonology (SIP).	Guideline
Contemporary narrative review of treatment options for COVID-19.	Review
Current status of therapeutic alternatives for COVID-19: A narrative review.	Review
Efficacy of COVID-19 Treatments: A Bayesian Network Meta-Analysis of Randomized Controlled Trials.	Review
Immunomodulatory therapies for the treatment of SARS-CoV-2 infection: an update of the systematic literature review to inform EULAR points to consider.	Review
Immunomodulatory therapies for SARS-CoV-2 infection: a systematic literature review to inform EULAR points to consider.	Review
Repurposed immunomodulatory drugs for Covid-19 in pre-ICu patients - mulTi-Arm Therapeutic study in pre-ICu patients admitted with Covid-19 - Repurposed Drugs (TACTIC-R): A structured summary of a study protocol for a randomised con	Protoco
Safety of nontumor necrosis factor-targeted biologics in the COVID-19 pandemic.	Non-RCTs
The use of Janus kinase inhibitors in the time of severe acute respiratory syndrome coronavirus 2 (SARS-CoV-2).	Review
Therapeutic role of immunomodulators during the COVID-19 pandemic- a narrative review.	Review
**ScienceDirect (Total n=4) (0 duplicate) (Total=4)**	
Baricitinib for patients with severe COVID-19—time to change the standard of care?	Review
Baricitinib: the first immunomodulatory treatment to reduce COVID-19 mortality in a placebo-controlled trial	Comment
Efficacy and safety of baricitinib for the treatment of hospitalised adults with COVID-19 (COV-BARRIER): a randomised, double-blind, parallel-group, placebo-controlled phase 3 trial	***Included***
Efficacy and safety of baricitinib plus standard of care for the treatment of critically ill hospitalised adults with COVID-19 on invasive mechanical ventilation or extracorporeal membrane oxygenation:	***Included***
**Scopus (Total n=17) (n=3 duplicates) (Total=14)**	
A Phase I/II Clinical Trial to evaluate the efficacy of baricitinib to prevent respiratory insufficiency progression in onco-hematological patients affected with COVID19: A structured summary of a study protocol for a randomised controlled trial	Protoco
Association and pharmacological synergism of the triple drug therapy baricitinib/remdesivir/rhACE2 for the management of COVID-19 infection	In vitro
Baricitinib plus remdesivir for hospitalized adults with Covid-19	***Included***
Clinical efficacy and safety of Janus kinase inhibitors for COVID-19: A systematic review and meta-analysis of randomized controlled trials	Review
Efficacy and Safety of Immunomodulators in Patients with COVID-19: A Systematic Review and Network Meta-Analysis of Randomized Controlled Trials	Review
Immune Therapy, or Antiviral Therapy, or Both for COVID-19: A Systematic Review	Review
JAK inhibition reduces SARS-CoV-2 liver infectivity and modulates inflammatory responses to reduce morbidity and mortality	Non-RCTs
Janus kinase inhibitors and major COVID-19 outcomes: time to forget the two faces of Janus! A meta-analysis of randomized controlled trials	Review
Janus Kinase inhibitors for the treatment of hospitalized patients with COVID-19	Review
Role of Low-Molecular-Weight Heparin in Hospitalized Patients with Severe Acute Respiratory Syndrome Coronavirus 2 Pneumonia: A Prospective Observational Study	Non-RCTs
The effect of drugs used in rheumatology for treating SARS-CoV2 infection	Review
The Efficacy and Safety of Janus Kinase Inhibitors for Patients With COVID-19: A Living Systematic Review and Meta-Analysis	Review
The use of Janus Kinase inhibitors in hospitalized patients with COVID-19: Systematic review and meta-analysis	Review
Use of Baricitinib in Patients with Moderate to Severe Coronavirus Disease 2019	Non-RCTs
**MedRxiv (Total n=5) (n=2 duplicate) (Total=3)**	
Baricitinib in patients admitted to hospital with COVID-19 (RECOVERY): a randomised, controlled, open-label, platform trial and updated meta-analysis	***Included***
Combined administration of inhaled DNase, baricitinib and tocilizumab as rescue treatment in COVID-19 patients with severe respiratory failure	Non-RCTs
Use of Baricitinib in Treatment of Covid-19: A Systematic Review	Review
**Reference list search (n=0) (Total=0)**	

Reason	Total (n)
**Review**	18
**Guideli**	2
**Letter**	1
**Update**	1
**Protoc**	2
**Comme**	1
**In vitro**	1
**Non-R**	6
Includ	**4**
**Exclud**	**32**

**Summary**	
**Total**	52
**Duplic**	16
**Exclud**	32
**Include**	4
