# Supplementary material for: Baricitinib for the Management of SARS-CoV-2-Infected Patients: A Systematic Review and Meta-Analysis of Randomised Controlled Trials
Source: Can J Infect Dis Med Microbiol. 2022 Aug 2;2022:8332819. doi: 10.1155/2022/8332819 (PMC9346539; doi:10.1155/2022/8332819)
Supplement: Supplementary Materials — The supplementary files are given separately as supplementary files S1 and S2. [file S2_file-Eli_Lilly.pdf › S2_file-Eli_Lilly.pdf]

0844150

January 20, 2022

Dear Dr. Sivananthan Manoharan,

Thank you for your request for information on Is it possible for me to know the exact number of events for IMV for treated and control groups and the number of participants in each group? It is straightforward for mortality (62 vs 100) but not for the primary outcome. In my paper, I am looking for mortality and disease progression -respiratory related functions (mainly IMV). It would be a great help if you could provide me with the number of events and participants for IMV from both groups., which was passed onto us by Cynthia Kartman. We appreciate the opportunity to be of service to you.

Thank you for this request. In the COV-BARRIER trial, patients may have progressed to invasive mechanical ventilation and varied in NIAID-OS score at different time points.

For this reason, the following analysis was conducted in patients with non-missing baseline and at least 1 post baseline observation.

Results are based on Day 1 through Day 28.

The number of patients who EVER progressed to invasive mechanical ventilation was

- 136/761 in the placebo group, and
- 125/764 in the baricitinib 4 mg group.

Among the patients who progressed to invasive mechanical ventilation (NIAID-OS 7)

- 76 patients died in the placebo group, and
- 47 patients died in the baricitinib 4 mg group.

Among the patients who never progressed to mechanical ventilation by Day 28 (N=625 placebo, N = 639 Baricitinib)

- 24 patients died in the placebo group, and
- 15 patients died in the baricitinib 4 mg group

0844150

This information is provided as a professional service in response to your unsolicited inquiry. It is intended to provide you with a fair, balanced, and objective review of the available scientific literature and/or data that you requested. This response is not intended to offer recommendations for use of this or any product inconsistent with approved product labeling. This information is not medical advice nor opinion regarding the patient's condition, health, or treatment. Each individual's health, condition and treatment therapies are unique and best assessed by their healthcare practitioner.

The data may contain information that may or may not completely match the current local prescribing information. It is the responsibility of the prescriber to use any product mentioned herein in accordance with the local label and his/her own clinical judgment. Kindly refer to the approved local package insert for locally approved indications and full prescribing information, copies of which are enclosed. If an article is provided, no additional reproduction or distribution is authorized. It is to be used for educational purposes only and is covered by local and international copyright laws.

Should you have any further queries or comments, please contact us at ASEAN_MEDINFOQUERIES@lilly.com.

Yours sincerely

Medical Information Desk
Eli Lilly and Company (India) Pvt. Ltd.

ENCLOSURES:

Package Insert
